# Supplementary material for: Enhancing the accuracy of register‐based metrics: Comparing methods for handling overlapping psychiatric register entries in Finnish healthcare registers
Source: Int J Methods Psychiatr Res. 2024 Jun 17;33(2):e2029. doi: 10.1002/mpr.2029 (PMC11181770; doi:10.1002/mpr.2029)
Supplement: Supplementary file 1 — Supporting Information S1 [file MPR-33-e2029-s001.docx]

Supporting information: Variables in the register before processing

Variables in the Finnish healthcare registers that are relevant for this methodology are presented in the tables below (as provided for this project).

More information on the registers is available on:

- the readme of the scripts of the current methodology at <https://github.com/kmmsks/hilmo_identify_episodes/>,
- the web pages of the Finnish Insitute for Health and Welfare:
  - <https://thl.fi/en/statistics-and-data/data-and-services/register-descriptions/care-register-for-health-care> and
  - <https://thl.fi/en/statistics-and-data/data-and-services/register-descriptions/register-of-primary-health-care-visits>,
- the national code server at <https://koodistopalvelu.kanta.fi/codeserver/>,
- the register handbooks (in Finnish only) at: <https://thl.fi/tilastot-ja-data/ohjeet-tietojen-toimittamiseen/hoitoilmoitusjarjestelma-hilmo/hilmo-opas>
- until 1991, regulations regarding the registers were published in the general letters by the former Medical Board of Finland (Lääkintöhallitus, 1811–1991).

## Secondary care registers

### Years 1996-2020

#### Main variables

| Variable name (lowercase) | Variable type | Years | Description |
| --- | --- | --- | --- |
| shnro | character | 1996-2020 | Personal ID, pseudonymized. |
| isoid | character | 1996-2020 | Entry ID, for combining data across datasets. |
| vuosi | numeric | 1996-2020 | Year. The year of the entry. |
| ilaji | numeric | 1996-2020 | Type of entry; 1: discharge, 2: count of patients in the last day of the year. |
| tupva | character | 1996-2020 | Starting date of the entry. |
| lpvm | character | 1996-2020 | End date of the entry. |
| ea | character | 1996-2020 | Medical specialty. Psychiatric specialties; 70: psychiatry (1994-present), 70F: geriatric psychiatry (1994-present), 70X: adolescent psychiatry (1994-2006), 70Z: forensic psychiatry (1994-present), 74: adolescent psychiatry (2003-present), 75: child psychiatry (1994-present), 75X: adolescent psychiatry (1994-2006). |
| paltu | numeric | 1996-2020 | A kind of service provider ID. |
| pala | numeric | 1996-2020 | Type of service; 1: inpatient care, 2: day surgery, 5: substance use services, 6: rehabilitation facility, 83: day hospital, 91 emergency department visit, 92: first appointment in secondary outpatient services, 93 follow-up appointment in secondary outpatient services, 94 consultation in in secondary outpatient services. |
| yhteystapa | character | 2019-2020 | Mode of contact; R10: in-person visit, R20: home visit, R30 workplace visit, R41 visit elsewhere, R52: real-time virtual contact, R56 non-real-time virtual contact, R60 consultation between professionals, R71: meeting, R72: handling of matter when the patient is not present, R80: inpatient care, R90: other. |
| kiireellisyys | character | 2019-2020 | Urgency; E: non-urgent, 3: inpatient care that has started urgently, 4: inpatient care that has started non-urgently, 5: acute appointment, 6: emergency department visit. |

#### Diagnoses (separate datasets)

| Variable name (lowercase) | Variable type | Description |
| --- | --- | --- |
| isoid | character | Entry ID, for combining data across datasets. |
| kentta | character | Field; PDGE: main diagnosis - cause, PDGO main diagnosis - symptom (ICD-10 combined codes), SDGE: additional diagnosis - cause, SDGO; additional diagnosis - symptom (ICD-10 combined codes), TAPTYYP: accident type, ULKSYY: external cause. |
| n | numeric | Number of the code in the field. |
| koodi | character | ICD-10 code. Point excluded from the code. |
| ulksyy | character | CD-10 code. Point excluded from the code. |

### Years 1994-1995

| Variable name (lowercase) | Variable type | Description |
| --- | --- | --- |
| shnro | character | Personal ID, pseudonymized. |
| tulopvm | character | Starting date of the entry. |
| lahtopvm | character | End date of the entry. |
| ea | character | Medical specialty. Psychiatric specialties; 70: psychiatry (1994-present), 70F: geriatric psychiatry (1994-present), 70X: adolescent psychiatry (1994-2006), 70Z: forensic psychiatry (1994-present), 74: adolescent psychiatry (2003-present), 75: child psychiatry (1994-present), 75X: adolescent psychiatry (1994-2006). |
| paltu | numeric | A kind of service provider ID. |
| pala | numeric | Type of service; 1: inpatient care, 2: day surgery, 5: substance use services, 6: rehabilitation facility, 83: day hospital, 91 emergency department visit, 92: first appointment in secondary outpatient services, 93 follow-up appointment in secondary outpatient services, 94 consultation in in secondary outpatient services. |
| pdg | character | Main diagnosis. |
| sdg1 | character | Additional diagnosis 1. |
| sdg2 | character | Additional diagnosis 2. |
| pitk | character | Long-term diagnoses. |

### Years 1987-1993

| Variable name (lowercase) | Variable type | Description |
| --- | --- | --- |
| shnro | character | Personal ID, pseudonymized. |
| vuosi | character | Year. The year of the entry. |
| tulopv | character | Starting date of the entry. |
| lahtopv | character | End date of the entry. |
| ea | character | Medical specialty; Psychiatric specialties; 70: psychiatry, 74: adolescent psychiatry, 75: child psychiatry. |
| pdg | character | Main diagnosis. |
| sdg1 | character | Additional diagnosis 1. |
| sdg2 | character | Additional diagnosis 2. |
| sdg3 | character | Additional diagnosis 3. |

### Years 1975-1986

| Variable name (lowercase) | Variable type | Description |
| --- | --- | --- |
| shnro | character | Personal ID, pseudonymized. |
| REKVV | character | Year. The year of the entry. |
| tulopvm | character | Starting date of the entry. |
| lahtopvm | character | End date of the entry. |
| ST | character | Hospital type.* |
| EALA | character | Medical specialty.* |
| DG1 | character | Diagnosis 1. |
| DG2 | character | Diagnosis 2. |
| DG3 | character | Diagnosis 3. |
| DG4 | character | Diagnosis 4. |

* psychiatric hospitalization:

- st: ‘mp’, OR
- st: ‘ys’ AND years 1976-1979 AND ea is in ‘70’, ‘71’, ‘72’, ‘73’, ‘74’, or ‘42’, OR
- st: ‘ys’ AND years 1980-1985 AND ea is in ‘70’, ‘71’, ‘72’, ‘73’, or ‘74’, OR
- years 1986-1986 AND ea is in ‘70’, ‘74’, or ‘75’.

See general letters N:o 1449, 1497, 1649, 1695, and 1877 by Lääkintöhallitus.

## Primary care registers 2011-2020

#### Main variables

| Variable name (lowercase) | Variable type | Description |
| --- | --- | --- |
| shnro |  | Personal ID, pseudonymized. |
| tapahtuma_tunnus |  | Entry ID, for combining data across datasets. |
| kaynti_palvelumuoto |  | Mode of appointment; |
| kaynti_yhteystapa |  | Type of contact; |
| kaynti_alkoi |  | Starting date of the entry. |
| kaynti_loppui |  | End date of the entry. |

#### Diagnoses (separate datasets)

| Variable name (lowercase) | Variable type | Description |
| --- | --- | --- |
| isoid | character | Entry ID, for combining data across datasets. |
| icd10 | character | ICPC-2 code. Point included in the codes. |
| icpc2 | character | ICPC-2 code. |
